# Is the Placenta an Immune Battlefield in Oocyte Donation? Histological Evidence of Graft-Versus-Host-like Phenomena in Triplet Pregnancies and the Development of PARS (Placental Allograft Rejection-like Score)

**DOI:** 10.3390/biomedicines14050965

**Published:** 2026-04-23

**Authors:** Eva Manuela Pena-Burgos, Jose Juan Pozo-Kreilinger, Rita María Regojo-Zapata, María De La Calle

**Affiliations:** 1 Pathology Department, Gregorio Marañón General University Hospital, Calle del Doctor Esquerdo 46, 28009 Madrid, Spain; 2 Pathology Department, La Paz University Hospital, 28046 Madrid, Spain; 3 Obstetrics and Gynecology Department, La Paz University Hospital, 28046 Madrid, Spain

**Keywords:** placenta, triplet pregnancy, oocyte donation, immune dysregulation, graft-versus-host-like, placental allograft rejection-like score

## Abstract

**Background/Objectives**: Oocyte donation (OD) pregnancies involve complete maternal–fetal genetic disparity and are associated with increased placental dysfunction and adverse perinatal outcomes. However, a unified histopathological framework to characterize alloimmune-mediated placental injury in OD gestations is lacking. This study evaluates immune and vascular alterations in OD triplet placentas and proposes a structured scoring system, the Placental Allograft Rejection-like Score (PARS), to quantify immunovascular dysregulation. **Methods**: This retrospective study included all OD triplet pregnancies with placental examination performed during 24 years at a tertiary referral center. Maternal, obstetric, fetal, neonatal, and pathological variables were analyzed at the pregnancy level. Histological and immunohistochemical features previously shown to differ between OD and non-OD pregnancies were grouped into six domains: innate immunity, adaptive immunity, checkpoint regulation, vascular remodeling, complement activation, and trophoblastic behavior. Binary thresholds, immunoreactive scores or established morphological cut-offs, were applied to construct a 20-point score classified into three grades. **Results**: Forty-five OD triplet pregnancies were analyzed. Intra-pregnancy concordance for PARS components was high, with intraclass correlation coefficient ≥0.70 in 87.3% pregnancies. Increasing PARS grades demonstrated a clear clinical gradient. Grade 3 pregnancies had significantly lower birthweight, higher rates of prematurity (<34 weeks), and increased fetal growth restriction. Placental weight decreased progressively with higher PARS. Histologically, grade 3 placentas showed significantly increased accelerated villous maturation and intervillous fibrin deposition. **Conclusions**: PARS integrates immune and vascular placental lesions into a structured and reproducible framework that correlates with clinically relevant perinatal outcomes and may support future clinical risk stratification, although further validation in larger, multicenter prospective cohorts is required.

## 1. Introduction

The placenta plays a critical role in mediating immune tolerance between the mother and the allogeneic fetus. In oocyte donation (OD) pregnancies, however, this balance becomes particularly challenging due to the complete genetic disparity between the embryo and the gestational carrier, resembling the conditions seen in solid organ transplantation [1]. OD pregnancies have been consistently associated with increased risks of preeclampsia and gestational hypertension [2,3,4,5,6,7], as well as preterm birth, low birthweight [5,8,9], and placental dysfunction [10,11] in singleton and twin gestations. In a recent study conducted in our population of triplet pregnancies, we found higher rates of pregnancy-induced hypertension, preeclampsia, fetal growth restriction (FGR), and fetal death in the OD group compared with non-OD assisted reproductive technology (ART) pregnancies [12].

Although the mechanisms underlying these clinical and pathological alterations remain poorly understood, immune and vascular dysfunction are thought to play central roles [10]. Several histopathological and immunohistochemical studies have identified key alterations in OD placentas, including increased infiltration of CD14^+^ and HLA-DR^+^ immune cells, reduced CD163^+^ regulatory macrophages, impaired vascular remodeling, and evidence of complement activation [13,14,15,16]. In our previous work in triplet pregnancies, OD placentas demonstrated reduced CD163^+^ macrophages, increased CD14^+^ and HLA-DR^+^ immune cells, and moderate PD-L1 expression in the decidua. CD4^+^ regulatory T cells were scanty, and CD8^+^ T cells were scarce. Vascular analysis revealed persistence of the muscular layer in maternal vessels, perivascular immune infiltration, and endothelial activation (WT1^+^, CD15^+^) in the absence of VEGF expression. Complement activation, reflected by increased C4d deposition, was also more frequent in the OD group [17]. Together, these findings resemble—within the placenta—the immunological mechanisms observed in graft-versus-host disease and antibody-mediated rejection. The concept of a graft-versus-host-like phenomenon in the placenta is emerging as a unifying model to explain immune-mediated placental dysfunction in highly allogeneic gestations.

Despite these insights, there is currently no standardized histopathological framework to quantify the severity of immune and vascular alterations in OD placentas. The absence of an integrative and predictive scoring system limits the translational value of placental pathology and its potential role in clinical risk stratification. The aim of this study is not only to characterize immune and vascular alterations in triplet OD placentas, but to integrate these findings into a structured, biologically grounded, and reproducible histopathological scoring system capable of capturing the global burden of alloimmune-mediated placental injury and its clinical correlates. By providing a unified framework, the score may facilitate interpretation, improve reproducibility, and support future research and clinical decision-making in pregnancies with high fetal allogenicity.

## 2. Materials and Methods

### 2.1. Study Design and Eligibility Criteria

All OD triplet pregnancies whose placentas were submitted for pathological evaluation at the Department of Pathology of our hospital between January 2000 and December 2024 were included. Clinical data were obtained from medical records of triplet pregnancies followed at the Maternal–Fetal Medicine Unit of La Paz University Hospital. The study was approved by the Research Ethics Committee of La Paz University Hospital (PI-5286, 2022.168; 14 July 2022). Triplet pregnancy diagnosis, chorionicity, amnionicity, and gestational age were determined by expert obstetric sonographers during the first trimester, and chorionicity was confirmed postnatally.

Unlike our previous study—where cases with well-defined etiologies (e.g., preeclampsia) or additional placental lesions such as villitis were excluded to isolate the immunological signature of OD—these cases were intentionally included in the present analysis. This broader inclusion strategy was chosen to capture the full histological heterogeneity of OD triplet placentas and to ensure that the development of the PARS reflects the real-world spectrum of immunovascular injury in highly allogeneic gestations. All OD triplet pregnancies were included regardless of pregnancy outcomes, gross findings, or histological lesions, except for deliveries before 28 weeks, which were excluded due to insufficient placental maturation. Cases with all live births and those with one or two stillbirths were grouped together for analysis within each study group. None of the patients included had immunosuppressive conditions, received immunosuppressive therapy, or had a diagnosis of autoimmune disease during pregnancy. Details regarding ART procedures, ovarian stimulation protocols, embryo culture, and oocyte donor selection have been extensively described previously [17].

### 2.2. Basal Study Variables

Baseline data included chorionicity, maternal age at conception, pre-pregnancy body mass index (BMI), year of conception, preexisting medical conditions associated with subfertility, previous failed pregnancy attempts (including previable intrauterine losses), and parity.

### 2.3. Maternal, Obstetric and Neonatal Outcomes

Maternal outcomes evaluated in this cohort included hypertensive disorders of pregnancy (preeclampsia and pregnancy-induced hypertension), gestational diabetes, intrahepatic cholestasis, pregnancy-related thyroid dysfunction, and iron-deficiency anemia. Fetal complications comprised FGR, twin-to-twin transfusion syndrome, twin anemia–polycythemia sequence, biometric or amniotic-fluid discordance, congenital anomalies, and intrauterine fetal death. Obstetric variables of interest included threatened preterm labor, premature rupture of membranes, and prematurity (<34 weeks). Perinatal outcomes included neonatal death, birthweight, umbilical cord pH, 5 min Apgar score, neonatal intensive care unit (NICU) admission, neonatal sepsis, respiratory distress syndrome, necrotizing enterocolitis, intraventricular hemorrhage, and retinopathy of prematurity. For quantitative neonatal variable—birthweight—a mean value was calculated from the three fetuses of each gestation, following the same approach used for quantitative placental variables. Fetal or neonatal events were considered present when identified in at least one fetus or neonate within the pregnancy.

### 2.4. Gross Placental Evaluation and Gross Variables

Gross placental findings were extracted from pathology reports. For quantitative measurements—placental weight, and placental thickness—the mean value of the three placental disks was used to represent each triplet gestation. Recorded macroscopic characteristics included umbilical cord insertion type (central, paracentral, marginal, or velamentous), cord thickness, and cord coiling pattern. Coiling was categorized as hypocoiled (<1 coil per 10 cm), normal (1–8 coils per 10 cm), or hypercoiled (>8 coils per 10 cm). The presence of hematomas (intraparenchymal, retroplacental, or subchorionic) was also documented. Placental weight was classified according to the percentile ranges established by Pinar et al. (≤10th, 11th–50th, 51st–89th, ≥90th percentiles) [18]. Deep vascular anastomoses were not assessed. Any gross abnormality was submitted for histopathological analysis.

### 2.5. Pathological Examination and Pathological Variables

All available placental material was re-evaluated, including hematoxylin–eosin–stained sections from umbilical cords, membranes, decidua, cord insertion sites, and villous parenchyma from each placental disk or sector according to chorionicity. For cases examined between 2000 and 2016, one villous parenchyma block per fetus was available, whereas from 2017 onward, two blocks per fetus were routinely analyzed. Tissue was fixed in 10% neutral buffered formalin for 24–48 h, embedded in paraffin, and sectioned at 5 μm. All samples were processed according to standardized institutional protocols for placental examination throughout the study period. For immunohistochemical analyses, staining procedures were performed using consistent protocols, and whenever possible, samples for each marker were processed in the same batch to minimize inter-assay variability. To assess the potential impact of long-term tissue storage on PARS scoring, a sensitivity analysis was performed. Cases were ordered chronologically according to year of conception and divided into quartiles. The oldest quartile and the most recent quartile were compared to evaluate whether archival time influenced PARS classification. Differences in PARS grade distribution between groups were analyzed using chi-square tests. All slides were reviewed by two pathologists with expertise in placental pathology, who assessed each case independently while blinded to PARS grade; discordant interpretations were resolved by consensus.

Histopathological findings were classified following the Amsterdam Placental Workshop Group Consensus and its updated definitions [19,20]. Lesions were grouped into three overarching patterns. The inflammatory pattern included acute chorioamnionitis (stages 2 and 3), acute funisitis (stages 1 to 3), chronic villitis of unknown etiology, chronic intervillositis, and chronic deciduitis. The vascular pattern comprised maternal vascular malperfusion (MVM)—including accelerated villous maturation, distal villous hypoplasia, and features of decidual arteriopathy—and fetal vascular malperfusion (FVM), such as vascular thrombi, avascular villi unrelated to villitis, stem vessel obliteration, stromal–vascular karyorrhexis, and intramural fibrin deposition. Other placental findings recorded were villous edema, chorangiosis, chorangioma, dystrophic calcifications, intervillous fibrin deposition, and the presence of nucleated red blood cells beyond 20 weeks’ gestation. For statistical purposes, all grades of acute chorioamnionitis, funisitis, and chronic villitis were analyzed collectively as single variables. Each lesion was considered present at the pregnancy level if identified in at least one placental disk within the gestation.

### 2.6. Histological Scale Construction

To develop a histopathological scoring system reflective of immune and vascular dysregulation in OD pregnancies, we used a previously characterized cohort of 115 triplet pregnancies—29 of them conceived through OD. These cases represent a model of maximal fetal allogenicity, central to the proposed concept of graft-versus-host-like placental phenomena. The conceptual structure of the score was inspired by the Banff classification used in renal transplantation [21], where morphological and immunohistochemical features are integrated to identify cellular and antibody-mediated rejection. By adapting this framework to placental pathology, our objective was to establish a reproducible system capable of capturing immune-mediated dysfunction in highly allogeneic gestations.

The score was applied at the pregnancy level. Each placental disk within triplet gestations was initially evaluated independently to assess potential intra-pregnancy variability. Concordance across the three disks was high, with intraclass correlation coefficients (ICC) ≥ 0.70 in 87.3% of pregnancies, indicating minimal within-gestation differences. Given this strong agreement—and to enhance clinical applicability—the PARS was ultimately defined as a single global score per pregnancy.

**Figure 1:**
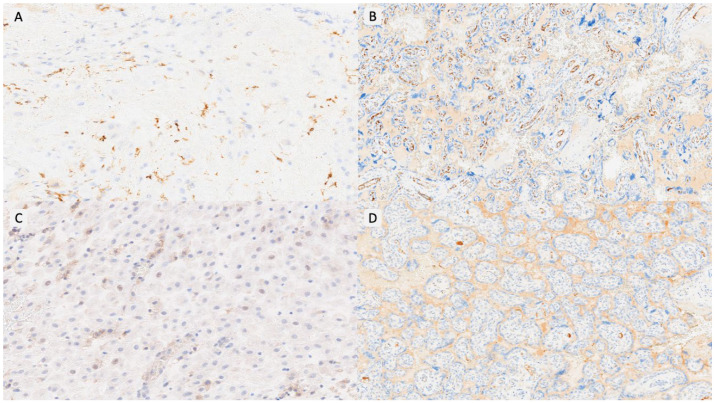
PARS Grade 1 example. (**A**). CD14/HLA-DR/NK-positive cells in decidua basalis (IRS < 8) (×100). (**B**). VEGF expression conserved in villous vessels (×40). (**C**). Absence of PD-L1 expression in decidua basalis cells (×200). (**D**). Absence of C4d deposition in villous basal membrane (×40).

**Figure 2:**
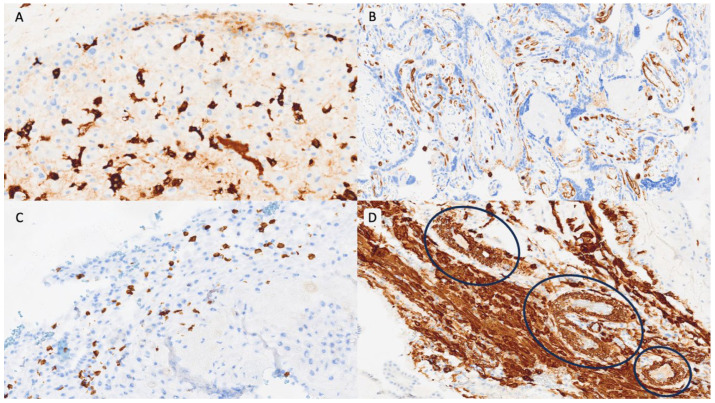
PARS Grade 2 example. (**A**). CD14/HLA-DR/NK-positive cells in decidua basalis (IRS < 8) (×100). (**B**). CD15 expression in villous vessels (×200). (**C**). CD8-positive T lymphocytes in decidua basalis (IRS < 8) (×100). (**D**). Smooth muscle presence in decidual vessels (smooth muscle actin-positive) (blue circles) (×200).

**Figure 3:**
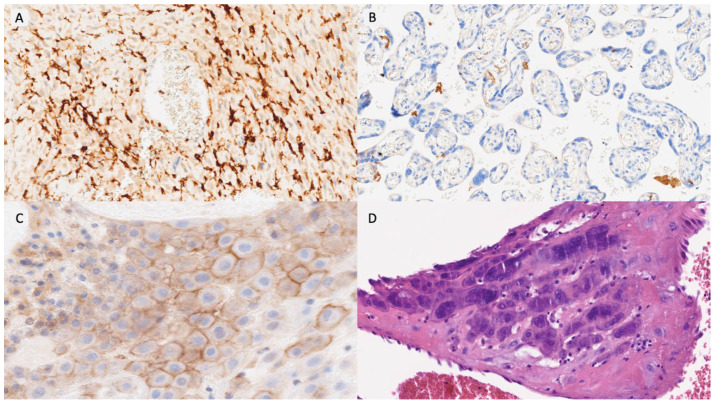
PARS Grade 3 example. (**A**). CD14/HLA-DR/NK-positive cells in decidua basalis (IRS > 8) (×100). (**B**). VEGF expression lost in villous vessels (×400). (**C**). PD-L1 expression in decidua (>50 cells/10 high power fields with complete and moderate or high staining (×400). (**D**). ≥Twenty multinucleated cells in decidua per-10 high power fields (hematoxylin and eosin, ×400).

Item selection was based on our previously published study of triplet placentas, which identified a characteristic immunovascular signature in OD pregnancies, including macrophage polarization, T-cell dysregulation, impaired vascular remodeling, and complement activation [17]. These variables were organized into six domains (Table 1): innate immunity, adaptive immunity, NK-cell activity, vascular remodeling, complement activation, and trophoblastic behavior. Each item was scored dichotomously (positive/negative) using predefined thresholds—most notably an immunoreactive score (IRS) ≥ 8 for immunomarkers and ≥20 multinucleated trophoblastic cells per 10 HPFs for morphological assessment. The detailed definition and calculation of all variables, including IRS methodology, are fully described in our previous publication [17]. Representative examples of each PARS grade are shown in Figure 1, Figure 2 and Figure 3.

The PARS was standardized to a 20-point semiquantitative scale. The 13 items contributed 1 or 2 points depending on biological significance, with IRS-based immune markers scored as binary variables (IRS ≥ 8 = positive) and composite morphological features more indicative of graft-versus-host-like phenomena weighted with 2 points. Based on the final score, placentas were classified into three categories: Grade 1 (PARS 0–6), Grade 2 (PARS 7–13), and Grade 3 (PARS 14–20). Individual PARS variables were not re-analyzed across PARS categories, as their thresholds, biological relevance, and reproducibility were validated in the foundational immunohistochemical study [17].

### 2.7. Statistical Analysis

Maternal, obstetric, neonatal, and placental outcomes were analyzed at the pregnancy level, with each triplet gestation contributing a single observation. Continuous variables were assessed for normality using the Kolmogorov–Smirnov test and expressed as mean ± standard deviation (SD) or median (interquartile range, IQR), as appropriate. Categorical variables were summarized as frequencies and percentages. Comparisons across the three PARS categories—Grade 1 (0–6), Grade 2 (7–13), and Grade 3 (14–20)—were performed using one-way ANOVA for normally distributed continuous variables or the Kruskal–Wallis test for non-normally distributed data. Categorical variables were compared using the chi-square test or Fisher’s exact test, as appropriate. When global significance was observed, post hoc pairwise analyses (Grade 1 vs. 2, 1 vs. 3, and 2 vs. 3) were carried out using the same statistical test applied in the global model. Intra-pregnancy concordance for PARS was assessed using the intraclass correlation coefficient (ICC), with ICC ≥ 0.70 indicating good agreement. Interobserver reliability between the two pathologists was also evaluated using ICC, with values > 0.75 considered acceptable. All analyses were performed using SPSS version 25 (IBM Corp., Armonk, NY, USA). Statistical significance was set at *p* < 0.05 (two-tailed). Given the exploratory nature of this study and the limited sample size inherent to triplet pregnancies, no correction for multiple comparisons was applied, following methodological recommendations for early-stage score development.

## 3. Results

### 3.1. General Results

**Figure 4:**
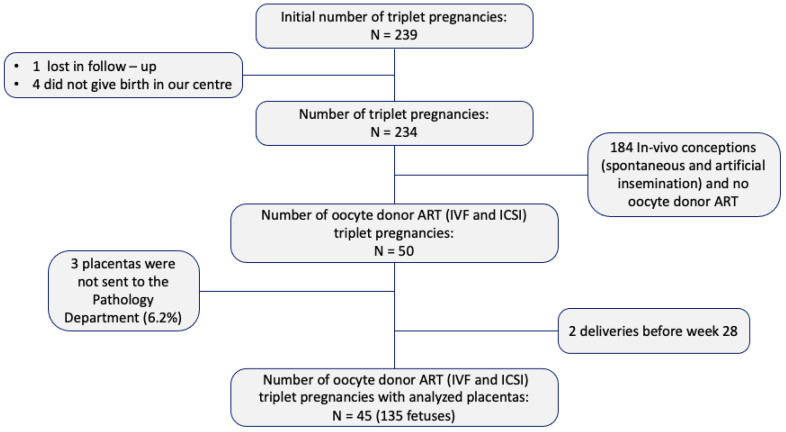
Inclusion and exclusion criteria. Number of placentas analyzed. ART: assisted reproductive technology; IVF: in vitro fertilization; ICSI: intracytoplasmic sperm injection.

A total of 239 women with triplet pregnancies were initially identified. Five women were excluded—one due to relocation during prenatal care and four who did not deliver at our institution. Among the remaining 234 pregnancies, 74 were conceived in vivo (spontaneously or via artificial insemination) and 110 through non-OD ART. Placental pathological examination was not performed in 3 cases (6.2%), and 2 additional pregnancies were excluded due to delivery before 28 weeks of gestation. Ultimately, 45 OD triplet pregnancies (135 fetuses) met the inclusion criteria and were analyzed (Figure 4).

### 3.2. Clinical Results

Only variables showing statistically significant differences across PARS categories are described below; all remaining results are provided in Table 2.

#### 3.2.1. Maternal Characteristics

Maternal age differed significantly across PARS categories (*p* = 0.04). Post hoc analyses showed that pregnancies with Grade 3 PARS were significantly older than those in Grade 1 (38.4 ± 2.5 vs. 36.2 ± 2.2 years; *p* = 0.022), whereas differences between Grade 1 vs. Grade 2 (*p* = 0.10) and Grade 2 vs. Grade 3 (*p* = 0.58) were not statistically significant. Pre-pregnancy BMI also varied significantly among groups (*p* = 0.02). Grade 3 pregnancies had a higher BMI compared with Grade 1 (26.2 ± 4.30 vs. 22.0 ± 2.35 kg/m^2^; *p* = 0.015), while the comparisons between Grade 1 vs. Grade 2 (*p* = 0.06) and Grade 2 vs. Grade 3 (*p* = 0.50) did not reach significance. Previous failed ART attempts were significantly associated with PARS category (*p* = 0.04), with the highest proportion observed in Grade 3 (58.8%), followed by Grade 2 (28.6%), and Grade 1 (20.0%).

#### 3.2.2. Fetal Complications

FGR showed a significant association with PARS grade (*p* = 0.02). Rates increased progressively from Grade 1 (0%) to Grade 2 (18.2%) and Grade 3 (42.9%). Post hoc comparison confirmed that Grade 3 had significantly higher FGR than Grade 1 (*p* = 0.01). Differences between Grade 1 vs. Grade 2 (*p* = 0.14) and Grade 2 vs. Grade 3 (*p* = 0.18) were not statistically significant.

#### 3.2.3. Obstetric Outcomes

Prematurity < 34 weeks differed significantly among groups (*p* = 0.02). Rates were 50.0% in Grade 1, 57.1% in Grade 2, and 90.5% in Grade 3. Post hoc analyses indicated significantly higher prematurity in Grade 3 compared with Grade 1 (*p* = 0.02). No significant differences were found for Grade 1 vs. Grade 2 (*p* = 0.72) or Grade 2 vs. Grade 3 (*p* = 0.07).

#### 3.2.4. Neonatal Outcomes

Birth weight differed markedly across PARS categories (*p* < 0.01), decreasing from Grade 1 (1984.5 ± 288 g) to Grade 2 (1322.2 ± 285.9 g) and Grade 3 (1253.9 ± 300.3 g). Post hoc analyses showed significant reductions in both Grade 1 vs. Grade 2 (*p* = 2.1 × 10^−5^) and Grade 1 vs. Grade 3 (*p* = 3.5 × 10^−6^), whereas no significant difference was found between Grade 2 vs. Grade 3 (*p* = 0.50). Birthweight < 1500 g also differed significantly (*p* < 0.01): 0% in Grade 1, 71.4% in Grade 2, and 85.7% in Grade 3. Post hoc analyses again showed strong differences between Grade 1 and both Grade 2 (*p* < 0.001) and Grade 3 (*p* < 0.001), while Grade 2 vs. Grade 3 remained non-significant (*p* = 0.28).

### 3.3. Gross and Histopathological Results

Only variables showing statistically significant differences across PARS categories are described below; all remaining results are provided in Table 3. A sensitivity analysis comparing the oldest and most recent quartiles showed no significant differences in the distribution of PARS grades (*p* = 0.11). The proportion of cases across PARS categories remained comparable between groups, with no evidence of a shift toward higher or lower scores in older samples.

#### 3.3.1. Gross Characteristics

Placental weight varied significantly across PARS categories (*p* = 0.01). Placentas in Grade 3 were substantially lighter (777.4 ± 217.9 g) compared with those in Grade 1 (1060.8 ± 259.2 g) and Grade 2 (1002.7 ± 227.3 g). Post hoc analyses confirmed that Grade 3 placentas were significantly lighter than both Grade 1 and Grade 2, whereas no difference was observed between Grades 1 and 2. A parallel trend was observed for placentas below the 10th percentile (*p* = 0.03): only 10.0% of Grade 1 placentas fell below this threshold, compared with 21.4% of Grade 2 and more than half of Grade 3 cases (52.4%). These findings suggest a stepwise reduction in placental mass accompanying higher PARS grades.

#### 3.3.2. Vascular Pattern Findings

Features of maternal vascular malperfusion showed a clear relationship with PARS severity. Accelerated villous maturation differed significantly among groups (*p* = 0.01), occurring in all Grade 3 placentas (100%), compared with 78.6% in Grade 2 and 60.0% in Grade 1. Post hoc comparisons indicated that the global significance was driven primarily by a marked difference between Grade 3 and Grade 1, with Grade 2 showing an intermediate, non-significant pattern.

#### 3.3.3. Other Histopathological Findings

Intervillous fibrin deposition was significantly associated with PARS category (*p* = 0.04), rising from 20.0% in Grade 1 and 21.4% in Grade 2 to 57.1% in Grade 3. Post hoc exploration confirmed that Grade 3 differed from Grades 1 and 2, which showed similar frequencies.

## 4. Discussion

### 4.1. Main Findings

In this study, we developed and applied the PARS to characterize the spectrum of immune and vascular dysregulation in triplet pregnancies conceived through OD. Our findings demonstrate that higher PARS grades are consistently associated with adverse maternal, fetal, and neonatal outcomes, supporting the concept that increasing immunovascular injury at the maternal–fetal interface contributes to clinically relevant placental dysfunction in highly allogeneic pregnancies. The strong associations between Grade 3 PARS and key complications—such as FGR, prematurity, and markedly reduced birthweight—highlight the potential of this scoring system to identify pregnancies at elevated risk of placental insufficiency. While individual histological and clinical variables may provide valuable information, their isolated interpretation may be fragmented and insufficient to capture the overall burden of placental immunovascular dysfunction. In contrast, PARS integrates these alterations into a structured and reproducible framework, allowing a more comprehensive and clinically meaningful assessment.

### 4.2. Clinical Results

Across PARS categories, a consistent clinical gradient emerged that supports the central hypothesis linking immune–vascular placental injury with adverse perinatal outcomes in OD triplet pregnancies. Maternal characteristics showed meaningful associations: women in the highest PARS category were older and had higher pre-pregnancy BMI, suggesting that maternal background may influence susceptibility to alloimmune dysregulation. The higher frequency of previous failed ART attempts in elevated PARS groups also raises the possibility—albeit speculative—of underlying reproductive or immunological factors contributing to placental maladaptation.

Neonatal outcomes provided the strongest evidence of clinical impact. Birthweight decreased sharply with increasing PARS, and extremely low birthweight (<1500 g) was almost entirely restricted to Grades 2 and 3. Prematurity followed a similar pattern, with Grade 3 showing a marked increase in deliveries before 34 weeks. Post hoc analyses identified the most pronounced differences between Grades 1 and 3, indicating that once histological injury surpasses a certain threshold, fetal compromise becomes consistently severe. These findings are biologically aligned with the histological phenotype of high PARS, characterized by impaired vascular remodeling, macrophage activation, and complement deposition—all processes capable of limiting placental perfusion and nutrient transport.

Several maternal and fetal complications—such as preeclampsia, gestational diabetes, or amniotic fluid discordance—did not reach statistical significance; however, their numerical increase in higher PARS categories, particularly the clustering of preeclampsia in Grade 3, supports a trend toward greater clinical instability. The lack of statistical significance is likely related to the modest sample size inherent to triplet gestations rather than absence of a biological association.

Altogether, the clinical patterns observed across the three PARS grades portray a continuum of graft-versus-host-like placental dysfunction, where escalating immunovascular injury translates into progressively worse perinatal outcomes. These findings underscore the potential of PARS as a prognostic indicator of placental insufficiency in high-allogenicity pregnancies, while highlighting the need for larger, prospective studies to validate its predictive utility and explore its integration into clinical decision-making.

### 4.3. Gross and Histopathological Results

The placental findings revealed a coherent and biologically plausible gradient of injury across PARS categories, supporting the internal validity of the scoring system. Grossly, Grade 3 placentas were markedly smaller, with over half falling below the 10th percentile. This progressive reduction in placental mass is consistent with chronic maternal underperfusion and impaired trophoblast function, reinforcing the link between increasing PARS severity and worsening placental efficiency.

Histologically, the vascular domain showed the strongest associations with PARS. Accelerated villous maturation and extensive intervillous fibrin deposition were all significantly more frequent in higher PARS grades. Together, these features reflect impaired spiral artery remodeling, sustained maternal malperfusion, and ongoing endothelial injury—hallmarks of severe immunovascular dysfunction. These findings align with the established immunological profile of OD placentas, where macrophage polarization, endothelial activation, and complement deposition create a microenvironment reminiscent of graft-versus-host–like pathology.

Several additional lesions, though not statistically significant, showed biologically meaningful trends that further support this continuum. Distal villous hypoplasia increased stepwise across PARS categories, mirroring the pattern of maternal vascular malperfusion. Stem vessel obliteration—found exclusively in Grade 3—suggests that only the most severe immunovascular dysregulation extends into the fetal circulation. Chorangiosis and villous edema also tended to cluster in higher grades, indicating chronic hypoxic stress. These patterns likely failed to reach statistical significance due to the limited sample size inherent to triplet pregnancies, but their consistency reinforces the plausibility of a dose–response relationship between immune dysregulation and placental injury. Not all lesions varied meaningfully across PARS categories, indicating that these features may represent background pathology of multifetal gestations rather than alloimmune-driven injury. Distinguishing PARS-related from non–PARS-related findings enhance the specificity of the score and prevents overinterpretation of unrelated histological noise.

Taken together, the gross and microscopic findings support the concept that high PARS identify placentas with substantial immunovascular compromise. The convergence of statistically significant results, consistent biological trends, and mechanistic plausibility demonstrates that PARS captures a meaningful gradient of alloimmune injury in OD triplet gestations. Importantly, whether these placental lesions—and the immunological dysregulation that underlies them—tend to recur in subsequent pregnancies remains unknown. Similar recurrence patterns have been reported in other immune-mediated placental disorders, including chronic inflammatory lesions [22], suggesting that future studies should examine the longitudinal reproducibility of PARS phenotypes across pregnancies. Overall, the histopathological data reinforce PARS as a promising framework for characterizing alloimmune-mediated placental dysfunction, providing a structured approach to quantify the immunovascular burden in highly allogeneic gestations.

### 4.4. Strengths and Limitations

The broader methodological strengths and limitations of this cohort—including the rarity of triplet gestations, the extensive histological and immunohistochemical characterization, and the reproducibility of IRS scoring—have been thoroughly detailed in our previous publication on immune and vascular alterations in OD placentas [17]. In the present study, our focus shifts to the specific strengths and limitations inherent to the development of the PARS.

A major strength of this work lies in the systematic and domain-based construction of the score, grounded in biologically meaningful items previously identified as altered in OD placentas. The integration of immune, vascular, and other markers into a unified framework provides a novel and conceptually coherent tool for characterizing alloimmune-mediated placental injury. The high intra-pregnancy concordance across placental disks supports the robustness of assigning one score per gestation, and the strong associations observed between higher PARS grades and clinically relevant outcomes further reinforce its potential applicability.

However, several limitations intrinsic to the creation of a new scoring system must be acknowledged. First, PARS was developed retrospectively and applied to post-delivery placental samples, preventing assessment of temporal dynamics or predictive capacity during pregnancy. Second, point allocation—although biologically justified—remains exploratory and requires external validation, ideally in independent cohorts and prospective designs. Third, the sample size, although substantial for triplet gestations, remains limited and should be acknowledged as a significant constraint. This limitation may affect the generalizability of the findings and restrict the ability to refine score thresholds or evaluate the independent contribution of each item. Fourth, IRS-based immunohistochemical scoring is semi-quantitative and subject to some degree of interobserver variability and would benefit from future incorporation of automated or digital quantification techniques. Fifth, more detailed neonatal outcome parameters, such as NICU length of stay, were not consistently available across the study period and therefore could not be reliably assessed. Future prospective studies should incorporate these variables to refine the clinical applicability and prognostic value of PARS. Finally, the long inclusion period may introduce potential selection bias and temporal variability in clinical management and tissue processing, which cannot be completely excluded, although no consistent trend suggesting histological degradation or temporal bias was observed.

### 4.5. Clinical Applicability and Future Directions

To our knowledge, this is the first study to systematize immune and vascular placental alterations in OD pregnancies through a structured histopathological scoring system. Although exploratory, PARS is grounded in consistent immunohistochemical and morphological findings, with point allocation based on biological plausibility, histological prominence, and anticipated clinical relevance. This framework provides a practical tool for characterizing alloimmune placental dysfunction and establishes the basis for future validation studies.

External validation of PARS in larger, multicenter cohorts represents a key next step to confirm its generalizability and clinical utility. In this context, validation in singleton OD pregnancies is particularly relevant. Given that triplet gestations represent a model of extreme fetal allogenicity, assessing whether similar immune–vascular signatures are present in singleton pregnancies will help clarify whether high PARS reflects a graded biological continuum or a threshold effect specific to high-order multiple gestations.

The potential clinical applications of PARS extend beyond descriptive pathology. Given the strong association between high PARSs and adverse outcomes, this scoring system could eventually help identify pregnancies at increased risk of placental insufficiency and guide closer surveillance or even targeted immunomodulatory strategies. Prospective studies are required to determine its predictive performance and therapeutic implications.

An additional question raised by our findings concerns the reproducibility of these placental immune signatures across gestations. Whether the immunovascular alterations captured by PARS represent a pregnancy-specific response or reflect a stable maternal predisposition remains unknown. Understanding whether high PARSs similarly recur across OD gestations will provide important insight into maternal alloimmune susceptibility and may improve counseling and risk assessment in future pregnancies.

## 5. Conclusions

The PARS provides a structured and biologically grounded framework to quantify immunovascular dysregulation in OD triplet placentas, offering a practical and reproducible tool to interpret placental pathology within a clinically meaningful context. Higher PARS grades were strongly associated with FGR, prematurity, and reduced placental mass, highlighting its potential utility as a marker of alloimmune-mediated placental dysfunction. Although validation in larger and prospective cohorts is required, PARS may ultimately serve as a risk-stratification instrument to support clinical decision-making in pregnancies with high fetal allogenicity.

## Figures and Tables

**Table 1:** The Placental Allograft Rejection-like Score (PARS) items description and scoring. The maximum score is 20 points and minimum is 0. Morphological and composite findings with higher diagnostic weight contribute 2 points. IRS: immunoreactivity score; HPF: high power field; MPF: medium power field.

Domain	Item	Scoring	Max Points
Innate immunity	CD14+ macrophages in decidua basalis (IRS ≥ 8)	IRS < 8 = 0 IRS ≥ 8 = 2	2
Innate immunity	CD163+ macrophages in decidua basalis (IRS < 8)	IRS ≥ 8 = 0 IRS < 8 = 2	2
Innate immunity	HLA-DR+ cells in decidua basalis (IRS ≥ 8)	IRS < 8 = 0 IRS ≥ 8 = 2	2
Adaptive immunity	Absence of T lymphocytes (CD4+) in decidua basalis (IRS < 8)	Absent = 0 Present = 1	1
Adaptive immunity	Presence of T lymphocytes (CD8+) (IRS ≥ 8) cells in decidua	Absent = 0 Present = 1	1
NK cells	CD56+ NK lymphocytes (IRS ≥ 8) in decidua basalis	IRS < 8 = 0 IRS ≥ 8 = 1	1
Checkpoint regulation	PD-L1 expression in decidual cells (>50 cells/10 HPF with complete and moderate or high staining)	Negative = 0 Positive = 2	2
Vascular	Smooth muscle presence in decidual vessels (five or more vessels with retained muscular walls in 10 HPF (400×))	Absent = 0 Present = 2	2
Vascular	WT1+ endothelial cells in villous vessels (consistent staining in at least one vessel per villi in 10 MPF (200×))	Negative = 0 Positive = 1	1
Vascular	VEGF absent in villous vessels (consistent absence in all vessels per villi in 10 MPF (200×))	Present = 0 Absent = 2	2
Vascular	CD15+ in villous vessels (consistent staining in at least one vessel per villi in 10 MPF (200×))	Negative = 0 Positive = 1	1
Complement	C4d deposition in villous basal membrane (positive when strong and continuous staining was present in more than 30% of the villi)	Negative = 0 Positive = 2	2
Trophoblast	≥20 multinucleated cells (CK7+, CD68−) per 10 HPFs	Absent = 0 Present = 1	1

**Table 2:** Description of maternal, obstetric, fetal and neonatal variables, statistical significance and factors associated with PARS grade. MCTA: monochorionic triamniotic; DCTA: dichorionic triamniotic; TCTA: trichorionic triamniotic; BMI: body mass index; ART: assisted reproductive technology. Maternal age: K-S test *p* = 0.26; BMI: K-S test *p* = 0.45; gestational age at delivery: K-S test *p* = 0.33; birth weight: K-S *p* = 0.12. Bold means statistically significant.

Variable	Total, n = 45	Grade 1 (PARS 0–6), n = 10	Grade 2 (PARS 7–13), n = 14	Grade 3 (PARS 14–20), n = 21	Signification (*p*)
**Chorionicity** – MCTA – DCTA – TCTA					0.65
	4 (8.9)	1 (10.0)	2 (14.3)	1 (4.8)	
	23 (51.1)	6 (14.3)	5 (35.7)	12 (57.1)	
	18 (40.9)	3 (4.8)	7 (50.0)	8 (38.1)	
**Maternal age (years)**	37.6 ± 2.6	36.2 ± 2.2	37.9 ± 2.7	38.4 ± 2.5	**0.04**
**Pre-pregnancy BMI (kg/m^2^)** – BMI ≥ 30	24.9 ± 4.13	22.0 ± 2.35	25.0 ± 4.01	26.2 ± 4.30	**0.02**
	6 (13.3)	0 (0)	2 (14.3)	4 (19.0)	0.34
**Year of conception** – 2000–2011 – 2012–2024					0.11
	14 (31.1)	1 (10.0)	7 (50.0)	6 (28.6)	
	31 (68.9)	9 (90.0)	7 (50.0)	15 (71.4)	
**Parity** – First gestation – Second or more gestation					0.90
	40 (88.9)	9 (90.0)	12 (85.7)	19 (90.5)	
	5 (11.1)	1 (10.0)	2 (14.3)	2 (9.5)	
**Previous failed pregnancy attempts by ART**	17 (37.8)	2 (20.0)	4 (28.6)	10 (58.8)	**0.04**
**Maternal complications**					
Preeclampsia	5 (11.1)	0 (0)	1 (7.1)	4 (19.0)	0.24
Pregnancy-induced hypertension	3 (6.7)	1 (10.0)	1 (7.1)	1 (4.8)	0.85
Gestational diabetes	7 (15.6)	0 (0)	3 (21.4)	4 (19.0)	0.30
Intrahepatic cholestasis	1 (2.2)	0 (0)	1 (7.1)	0 (0)	0.32
Pregnancy-induced hypothyroidism	5 (11.1)	0 (0)	1 (7.1)	4 (19.0)	0.24
Iron deficiency anemia	12 (26.7)	2 (20.0)	4 (28.6)	6 (28.6)	0.86
**Fetal complications**					
Twin-to-twin transfusion syndrome	3 (6.7)	0 (0)	0 (0)	3 (14.3)	0.15
Twin anemia-polycythemia sequence	0 (0)	0 (0)	0 (0)	0 (0)	-
Amniotic fluid discordance	2 (4.4)	1 (10.0)	0 (0)	1 (4.8)	0.50
Biometry discordance	4 (8.9)	1 (10.0)	0 (0)	3 (14.3)	0.34
Fetal growth restriction	11 (24.4)	0 (0.0)	2 (18.2)	9 (42.9)	**0.02**
Fetal malformations	1 (2.2)	0 (0)	0 (0.0)	1 (4.8)	0.55
Fetal death	5 (11.1)	0 (0)	1 (7.1)	4 (19.0)	0.24
**Obstetric complications**					
Threatened preterm labor	6 (13.3)	1 (10.0)	2 (14.3)	3 (14.3)	0.56
Premature rupture of membranes	4 (8.9)	1 (10.0)	2 (14.3)	1 (4.8)	
**Prematurity**	31.7 ± 2.4	32.9 ± 2.3	32.5 ± 2.0	30.6 ± 2.4	
Prematurity (<34 weeks) – <31.6 weeks	32 (71.1)	5 (50.0)	8 (57.1)	19 (90.5)	**0.02**
	22 (48.9)	3 (30.0)	5 (35.7)	14 (66.7)	0.08
**Neonatal complications**					
Birth weight (g) – <1500 g	876.0 ± 412.9	1984.5 ± 288.8	1322.2 ± 285.86	1253.9 ± 300.3	**<0.01**
	28 (62.2)	0 (0)	10 (71.4)	18 (85.7)	**<0.01**
Umbilical cord pH < 7.20	5 (11.1)	0 (0)	1 (7.1)	4 (19.0)	0.24
Apgar score 5 min ≤ 5	7 (15.6)	0 (0)	2 (14.3)	5 (23.8)	0.22
Neonatal death	2 (4.4)	0 (0)	0 (0)	2 (9.5)	0.30
Neonatal intensive care admission	11 (24.4)	2 (20.0)	3 (21.4)	6 (28.6)	0.83
Neonatal sepsis	4 (8.9)	1 (10.0)	1 (7.1)	2 (9.5)	0.96
Respiratory distress syndrome	4 (8.9)	0 (0)	1 (7.1)	3 (14.3)	0.41
Necrotizing enterocolitis	3 (6.7)	0 (0)	1 (7.1)	2 (9.5)	0.61
Intraventricular hemorrhage	6 (13.3)	0 (0)	2 (14.3)	4 (19.0)	0.34
Retinopathy of prematurity	3 (6.7)	0 (0)	1 (7.1)	2 (9.5)	0.61

**Table 3:** Description of gross and histopathological variables, statistical significance and factors associated with PARS grade. Placental weight: K-S test *p* = 0.41, placental thickness: K-S test *p* = 0.02; umbilical cord thickness: K-S test *p* = 0.03. Bold means statistically significant.

Variable	Total, n = 45	Grade 1 (PARS 0–6), n = 10	Grade 2 (PARS 7–13), n = 14	Grade 3 (PARS 14–20), n = 21	Signification (*p*)
**Placental weight (g)**	914.6 ± 255.8	1060.8 ± 259.2	1002.7 ± 227.3	777.4 ± 217.9	**0.01**
**Placental weight ≤ 10**	15 (33.3)	1 (10.0)	3 (21.4)	11 (52.4)	**0.03**
**Placental thickness (cm)**	2.1 (P25 = 2.0; P75 = 2.4)	2.1 (P25 = 2.0; P75 = 2.5)	2.2 (P25 = 2.0; P75 = 2.5)	2.2 (P25 = 2.0; P75 = 2.5)	0.64
**Abnormal umbilical cord insertion (marginal or velamentous)**	35 (77.8)	9 (90.0)	10 (71.4)	16 (76.2)	0.54
**Umbilical cord thickness**	1.2 (P25 = 1.0; P75 = 1.4)	1.2 (P25 = 1.0; P75 = 1.4)	1.2 (P25 = 1.0; P75 = 1.4)	1.2 (P25 = 1.0; P75 = 1.4)	0.91
**Umbilical cord coiling** – Hypocoiling (<1) – Hypercoiling (>8)	4 (8.9)	1 (10.0)	1 (7.1)	2 (9.5)	0.71
**Intraparenchymatous infarct/hematoma**	12 (26.7)	2 (20.0)	3 (21.4)	7 (33.3)	0.63
**Retroplacental hematoma**	11 (24.4)	2 (20.0)	3 (21.4)	6 (28.6)	0.83
**Subchorionic hematoma**	2 (4.4)	1 (10.0)	1 (7.1)	0 (0)	0.37
**Inflammatory pattern findings**	14 (31.8)	2 (20.0)	4 (28.6)	8 (31.8)	0.57
Acute chorioamnionitis	4 (8.9)	1 (10.0)	2 (14.3)	1 (4.8)	0.62
Acute funisitis	4 (8.9)	1 (10.0)	2 (14.3)	1 (4.8)	0.62
Chronic deciduitis	2 (4.4)	0 (0)	1 (7.1)	1 (4.8)	0.70
Chronic villitis of unknown etiology	3 (6.7)	0 (0)	1 (7.1)	2 (9.5)	0.60
Chronic intervillitis	1 (2.2)	0 (0)	0 (0)	1 (4.8)	0.55
**Vascular pattern findings**					
Maternal vascular malperfusion					
Accelerated villous maturation	38 (84.4)	6 (60.0)	11 (78.6)	21 (100)	**0.01**
Distal villous hypoplasia	27 (60.0)	3 (30.0)	10 (71.4)	14 (66.7)	0.08
Decidual arteriopathy	1 (2.2)	0 (0)	0 (0)	1 (4.8)	0.55
Fetal vascular malperfusion					
Vascular trombi	5 (11.1)	0 (0)	2 (14.3)	3 (14.3)	0.44
Avascular villi	7 (15.6)	0 (0)	3 (21.4)	4 (19.0)	0.30
Stem vessels obliteration	4 (8.9)	0 (0)	0 (0)	4 (19.0)	0.08
Stromal-vascular karyorrhexis	7 (15.6)	0 (0)	3 (21.4)	4 (19.0)	0.30
Intramural fibrin deposits	1 (2.2)	0 (0)	0 (0)	1 (4.8)	0.55
**Other findings**					
Villous edema	11 (24.4)	3 (30.0)	3 (21.4)	5 (23.8)	0.88
Chorangiosis	19 (42.2)	4 (40.0)	5 (35.7)	10 (47.6)	0.77
Chorangioma	3 (6.7)	1 (10.0)	1 (7.1)	1 (10.0)	0.85
Dystrophic calcifications	7 (15.6)	2 (20.0)	3 (21.4)	2 (9.5)	0.58
Intervillous fibrin deposits	17 (37.8)	2 (20.0)	3 (21.4)	12 (57.1)	**0.04**
Nucleated red blood cells	3 (6.7)	1 (10.0)	1 (7.1)	1 (10.0)	0.85

## Data Availability

The raw data supporting the conclusions of this article will be made available by the authors on request.
